# Facile Fabrication of Dual Functional Graphene Oxide Microcapsules Carrying Corrosion Inhibitor and Encapsulating Self-Healing Agent

**DOI:** 10.3390/polym14194067

**Published:** 2022-09-28

**Authors:** Jing Li, Zhenglin Tao, Jincan Cui, Shuling Shen, Hanxun Qiu

**Affiliations:** 1 School of Materials and Chemistry, University of Shanghai for Science and Technology, No. 516 Jungong Road, Shanghai 200093, China; 2 School of Mechanical Engineering, Nantong University, No. 9 Seyuan Road, Nantong 226019, China

**Keywords:** composite coatings, microcapsules, graphene oxide, corrosion protection, self-healing

## Abstract

Dual functional graphene oxide (GO) microcapsules were fabricated through self-assembly in Pickering emulsions, carrying corrosion inhibitor benzotriazole (BTA) on the microcapsule shells and encapsulating a self-healing agent epoxy monomer. The formation of the GO microcapsules was assisted by the interaction between BTA and GO, which provided robust encapsulation for the epoxy monomer. The loading capacity of BTA and epoxy monomer reached 90.5%. The addition of the GO microcapsules simultaneously promoted the corrosion protection and self-healing properties of the waterborne epoxy composite coatings. The healing efficiency of the composite coatings reached over 99.7% when the content of the microcapsules was 10 wt%. Meanwhile, the corrosion current density of the intact coatings was decreased for around 50 times.

## 1. Introduction

Microcapsule technology has been used in polymer matrix composites to impart functional properties such as self-healing, self-reporting, and corrosion inhibiting [1,2]. Functions of the microcapsules usually depend on the selection of the core materials, which are released upon environmental stimulations. The shell of the microcapsules provide the protection of core materials, an efficient response to triggering conditions, and a compatible interface between the microcapsules and polymer matrix [3,4].

The fabrication methods of microcapsules included in situ emulsion polymerization [5,6], solvent evaporation phase separation [7,8], and self-assembly in Pickering emulsions [9,10]. The former two methods were usually employed to prepare microcapsules with polymeric shells. In situ polymerization in oil/water emulsion was employed to produce microcapsules with shells such as polyurea-formaldehyde [11], polyurethane [12], polyurea [13], and phenol formaldehyde [14]. The solvent evaporation phase separation method can be applied to encapsulate hexadecane by a wide range of polymeric shells including poly(1-lactide), poly(methyl methacrylate), poly(vinyl formal), poly(vinyl acetate), poly(2,6-dimethyl-1,4-phenylene oxide), poly(vinyl cinnamate) [8], and polystyrene [15]. The phase separation during solvent evaporation was driven by the hydrophilicity difference between the polymeric shell and self-healing agent in the nanodroplets.

We proposed the fabrication of graphene oxide (GO) microcapsules through the self-assembly of GO at the oil/water interface in Pickering emulsions, where linseed oil was encapsulated as the healing agent [16,17]. According to Pieranski’s equation:

(1) $$\Delta E=\frac{-\pi r^{2}}{\gamma_{o/w}}{\left[\gamma_{o/w}-\left(\gamma_{p/w}-\gamma_{p/o}\right)\right]}^{2}\;$$

A higher decrease in the interfacial energy (Δ*E*) is beneficial for the stabilization of Pickering emulsions, where *r* is the radius of the solid particles at the oil/water interface; *γ_o/w_* is the surface tension of the oil phase with respect to water; *γ_p/o_* and *γ_p/w_* are the surface tension of the particles with respect to oil and water, respectively [18]. The amphiphilic properties of GO gave smaller *γ_p/w_−γ_p/o_*, which was favorable for the stabilization of Pickering emulsions. In addition, the high specific surface area and two-dimensional shape of GO made it a unique choice for the microcapsule shells. Shells with a nanometer thickness and the high loading capacity of microcapsules was obtained by GO microcapsules [16]. Furthermore, a higher *γ_o/w_* value gives a higher Δ*E*, which limited the selection of the core materials. High interfacial energy between linseed oil and water in Pickering emulsions favored the self-assembly of GO sheets at the oil/water interface. The application of GO microcapsules would be extended by encapsulating other healing agents for a versatile polymer matrix.

To fulfil multi-functions, either multilayer-microcapsules [19] or dual functional microcapsules [20] were prepared. Healing agent linseed oil and corrosion inhibitor benzotriazole (BTA) were stored separately in multi-layer microcapsules through in situ polymerization and layer-by-layer assembly [21]. The fabrication process was tedious and the loading capacity was limited by the multilayer-shells. Dual functional microcapsules were prepared to encapsulate the mixture of healing agent and color indicator [22,23,24], to impart self-healing and self-reporting properties.

Here, we report on the dual functional GO microcapsules carrying corrosion inhibitor BTA and encapsulating a self-healing agent epoxy monomer. GO microcapsules were facilely fabricated by the self-assembly of GO in Pickering emulsions. While the assembly of GO was at the oil/water interfaces, BTA molecules also migrated to the interface from the oil phase, driven by the relative high hydrophilicity of BTA. Then, BTA molecules interacted with GO at the microcapsule shells, which assisted in the formation of the GO microcapsules, so that the GO/BTA shells were not only encapsulating the healing agent, but also inhabiting the corrosion process. The dual functional GO microcapsules were applied in a waterborne epoxy matrix to realize self-healing and corrosion inhibiting properties.

## 2. Experimental

### 2.1. Materials

Epoxy monomer and curing agent (Araldite^®^ 2020A and 2020B, HUNTSMAN, Shanghai, China) were used as the healing agent. The weight ratio of 2020A/2020B was 100:33. The waterborne epoxy (WEP) coatings were composed of the waterborne emulsion and curing agent (STW703A and 703B, Huayi Fine Chemical, Shanghai, China). The weight ratio of 703A/703B was 100:14. An aqueous dispersion of GO (10 mg/mL, Angstron Materials, Xiamen, China) was diluted and sonicated before use. BTA (99%, Adamas, Shanghai, China) was used as-received.

### 2.2. Fabrication of the GO Microcapsules

Different amounts of BTA were mixed with 1.4 g 2020A by magnetic stirring for 10 min. The mixture was added into 5 mL GO aqueous solution (3.75 mg/mL). The Pickering emulsions were prepared upon high shear mixing at 12,000 rpm/min for 8 min. The resultant was filtered and washed with excess DI water to collect the GO microcapsules. The GO microcapsules were referred to as MC-0, 1, 2, 3, 4, 5, respectively, with an increasing content of BTA, as listed in Table 1.

### 2.3. Preparation of the Microcapsules Reinforced WEP Coatings

The GO microcapsules were dispersed in 703A. The proper amount of 703B and 2020B were added into the mixture. The wet coatings were applied on the hot-dip-galvanized steel (HDG) sheets by a bar coater (65#, RD Specialties, Inc., Webster, NY, USA) after the solid content was adjusted to 35 wt%. The GO microcapsules/WEP composite coatings were obtained after drying at 80 °C for 20 min.

### 2.4. Characterization

The chemical composition of the microcapsules was investigated by an X-ray photoelectron spectrometer (XPS, PHI5300 ESCA) and Fourier transform infrared spectroscopy (FTIR, Spectrum 100 PerkinElmer, MA, USA). Thermogravimetric analysis (TGA, Pyris I, PerkinElmer) was performed in the range from 50 °C to 500 °C at a rate of 10 °C min^−1^ under N_2_ protection. Scanning electron microscope (SEM, Quanta FEG450 FEI, OR, USA) characterized the morphology. Scratches were applied manually by a razor blade, perpendicular to the coating surfaces with around a 30 μm width, reaching the HDG substrates.

Tafel plots were obtained by an electrochemical workstation (PARSTAT 4000, Princeton) in a 3.5% NaCl solution from −100 mV to +100 mV (vs. OCP) at a rate of 1 mV s^−1^, where the working electrode was the coated HDG with an area of 1 cm^2^, the reference electrode was Ag/AgCl (0.205 V/SHE), and the counter electrode was a platinum net. The healing efficiency (HE) was calculated after the introduction of scratches by:

(2) $$HE\left(\%\right)=\frac{I_{corr}^{WEP}-I_{corr}^{MC}}{I_{corr}^{WEP}}\times 100\;$$

where $I_{corr}^{WEP}$ and $I_{corr}^{MC}$ were the corrosion current densities (*I_corr_*) for the specimens coated by the neat WEP and coatings with the GO microcapsules [25].

## 3. Results and Discussion

### 3.1. Formation of the GO Microcapsules with the Assistance of BTA

**Figure 1:**
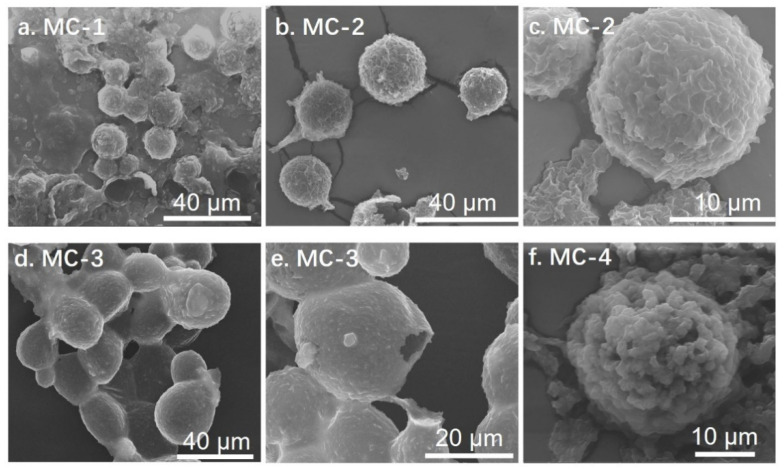
The morphology of the GO microcapsules from (**a**–**f**) MC-1 to MC-4 with an increasing content of BTA.

The integral GO microcapsules with spherical shape were observed for MC-2, MC-3, and MC-4, when the BTA content was intermediate, as shown in Figure 1. The formation of microcapsules failed for MC-0 and MC-5 without BTA and with an excessive amount of BTA, respectively. MC-1 was considered as the critical state, showing both collapsed and integral microcapsules (Figure 1a). The average diameter of the microcapsules increased from 12.5 μm to 17.2 μm with an increase in the BTA content (Table 1) by measuring five fields of SEM images with a magnification of around 3000 times. It seems that the addition of BTA played an important role in the formation of the GO microcapsules. The amount of BTA should be controlled between MC-2 and MC-4. The composition of MC-0, MC-1, and MC-5 did not produce integral microcapsules, so the corresponding average diameter and loading capacity were not provided in Table 1.

The surface morphology of the GO microcapsules was observed with a higher magnification, as shown in Figure 1c,e,f. The wrinkles on the surface of MC-2 resembled the morphology of GO. The self-assembly of GO at the oil/water interface in the Pickering emulsions led to the formation of microcapsule shells [16], driven by the decrease in the interfacial energy (Δ*E*) in Equation (1) [18]. The microcapsule surfaces became smooth for MC-3. The oil phase in the Pickering emulsions was the mixture of BTA and 2020A. The BTA molecules had higher hydrophilicity than 2020A, so BTA tended to migrate to the water/oil interfaces according to the phase separation principles [7]. Graphene-based materials had strong absorption properties [26]. As a result, BTA promoted the formation of the GO microcapsules by interacting with GO at the interfaces, which in turn provided a robust encapsulation of the core materials. With an increase in the BTA content, the wrinkles of GO were covered for MC-3. The accumulation of BTA at the microcapsule surfaces resulted in the rough surfaces for MC-4. When the content of BTA was further increased, the Pickering emulsions were no longer stable due to the decreased *γ_o/w_* in Equation (1) [18]. In contrast, the microcapsules cannot be formed for MC-0 without the assistance of BTA.

**Figure 2:**
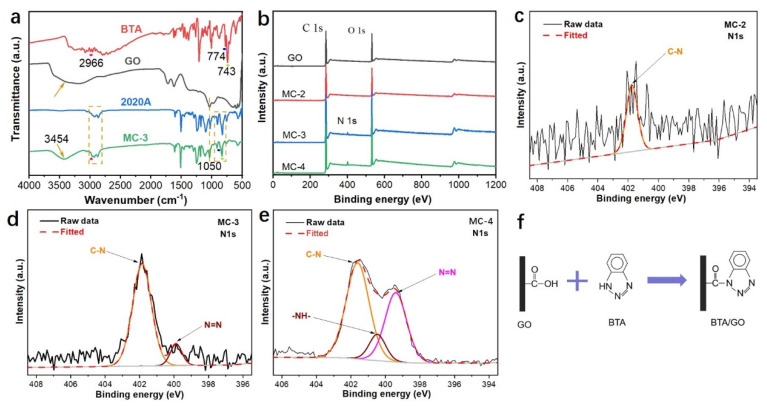
The FTIR (**a**) and XPS (**b**) spectra of the GO microcapsules. High-resolution XPS scans of N1s for MC-2 (**c**), MC-3 (**d**), and MC-4 (**e**). Reaction scheme of GO and BTA (**f**).

The FTIR spectrum of MC-3 inherited most of the peaks from 2020A, as shown in Figure 2a, because 2020A was encapsulated and dominated the composition of GO microcapsules. The peaks at 743 cm^−1^, 774 cm^−1^, and 2966 cm^−1^ belong to the –NH bending vibrations and the –CH stretching vibrations of BTA. The broader peak at 3454 cm^−1^ came from the –OH stretching vibration of GO. These peaks were also inherited by MC-3, suggesting the GO microcapsules were composed of 2020A, BTA, and GO.

The XPS spectra of the GO microcapsules (Figure 2b) showed that the N atomic content was increased from 0.7% to 3.62% with an increase in the BTA content (Table 2). The N1s peaks were deconvoluted to analyze the interaction between BTA and GO at the interfaces, as shown in Figure 2c–e. The nitrogen-containing groups in BTA were C–N, N=N, and NH. The N1s peaks of MC-2 and MC-3 were dominated by the C–N peak with a very small N=N peak, while the NH groups of BTA disappeared, which indicated the amidation reaction between the –NH groups of BTA and the –COOH groups of GO, as schematically illustrated in Figure 2f. With an increase in the BTA content, the peaks of N1s for MC-4 changed from a single peak to a doublet, which can be fitted into C–N, NH, and N=N components. The presence of the NH component suggested the amount of BTA in MC-4 was excessive after the amidation reaction.

**Figure 3:**
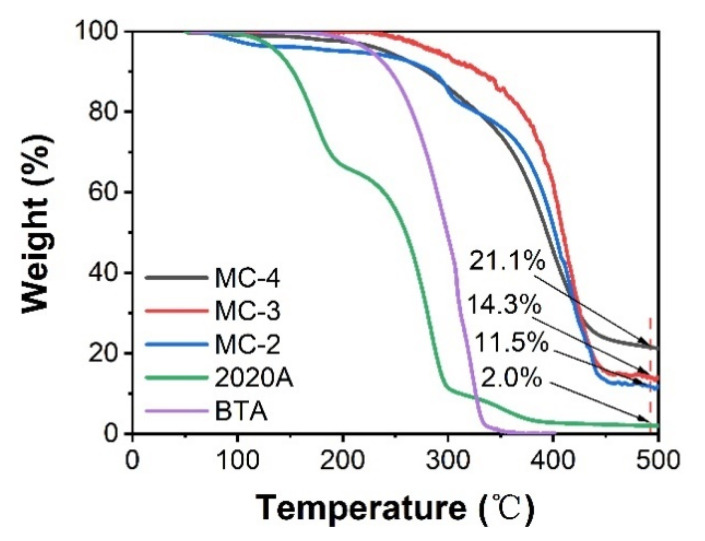
The TGA spectra of the BTA, 2020A, and GO microcapsules.

The loading capacity of the GO microcapsules were evaluated according to the TGA results in Figure 3. The decomposition of 2020A started at around 120 °C with a residual weight of 2.0% upon heating to 500 °C. BTA barely showed any residual weight at above 370 °C. The loading capacity of the MC-2 was estimated as: 1 − (11.5% − 2.0%) = 90.5%, neglecting the weight loss from the thermal reduction of GO. Similarly, the loading capacity of MC-3 and MC-4 was calculated and listed in Table 1. With an increase in the BTA content, the loading capacity of the GO microcapsules was slightly decreased. It was speculated that the amount of entrapped GO layers at the interface would increase with the BTA content, resulting in the slight decrease in the loading capacity as well as the increase in the microcapsule diameter.

**Figure 4:**
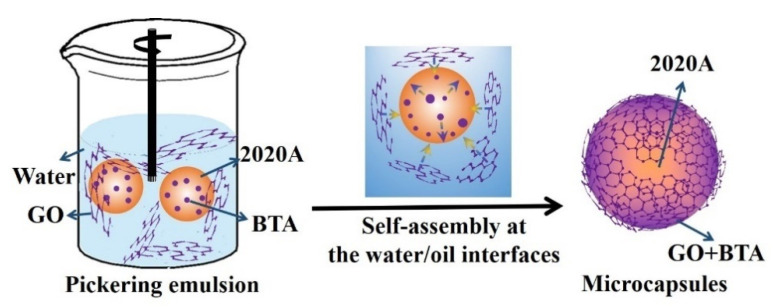
The formation schematics of the GO microcapsules.

The formation process of the GO microcapsules is illustrated in Figure 4. GO was dispersed in the water phase, while BTA was dispersed in 2020A (oil phase). Upon high shear mixing, the Pickering emulsions were obtained when the GO layers assembled spontaneously at the oil/water interface due to the amphiphilic feature of GO. Meanwhile, BTA molecules also migrated to the interface from the oil phase, driven by the relatively high hydrophilicity of BTA, compared to 2020A. Then, the amidation reaction (Figure 2f) occurred between GO and BTA, which assisted the robust encapsulation of the self-healing agent. An excessive amount of BTA can be carried physically on the microcapsule shells, as proven by Figure 1f and Figure 2e. The physical and chemical interactions between BTA and GO provided the microcapsule shells with the corrosion inhibition properties, so the microcapsules can be considered as a unique albumen-yolk-like structure.

### 3.2. Corrosion Protection and Self-Healing Properties of the Composite Coatings

**Figure 5:**
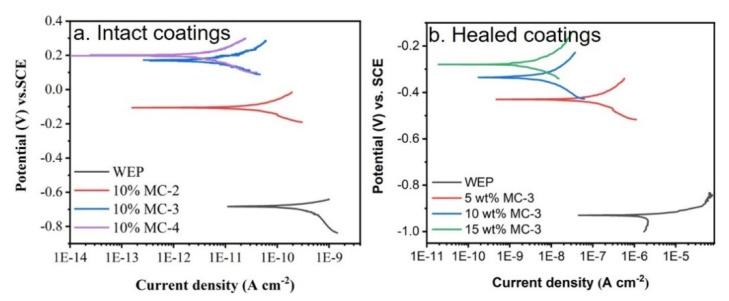
The Tafel plots of intact coatings containing 10 wt% of different GO microcapsules (**a**) and healed coatings containing different contents of MC-3 (**b**).

The corrosion protection properties of the composite coatings containing 10% GO microcapsules were significantly higher than that of the neat WEP coatings, as shown in Figure 5a. The corresponding *I_corr_* values, as listed in Table 3, decreased with the BTA content in the GO microcapsules from MC-2 to MC-4. BTA molecules, carried on the shells of GO microcapsules, can react with metal ions (Zn^2+^, Fe^2+^) and form a compacted complex, which inhibited the corrosion process of metals [27,28]. With the addition of 10 wt% MC-3 or MC-4, the *I_corr_* values were decreased for about two orders of magnitudes compared with the neat WEP coatings.

**Figure 6:**
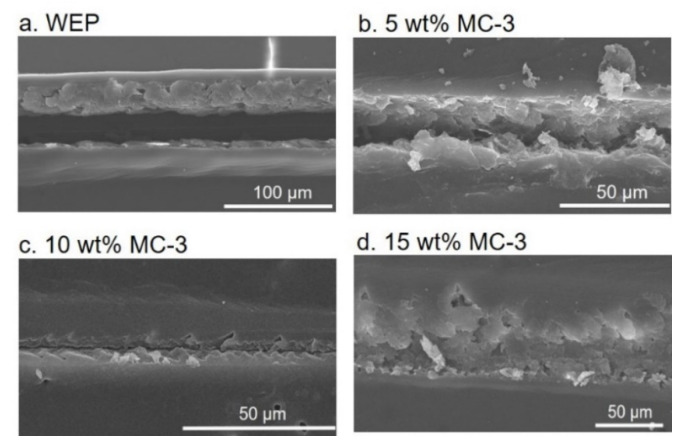
The morphology of the healed coatings containing (**a**) 0%, (**b**) 5 wt%, (**c**) 10 wt% and (**d**) 15 wt% of MC-3.

After scratching, the *I_corr_* value of WEP coatings increased to 1.68 × 10^−6^ A cm^−2^. The composite coatings with different contents of MC-3 were healed after 48 h (Figure 5b). The *I_corr_* values decreased with an increase in the content of MC-3 (Table 3) and the HE was calculated as 99.7% and 99.9% according to Equation (2), respectively, when the content of MC-3 was 10 wt% and 15 wt%. Figure 6 shows the morphology of the healed coatings. The release of the healing agent 2020A was triggered by crack-initiation and the cracks with a width of around 30 μm were filled up when the content of MC-3 was above 10 wt%, which was consistent with the results of the Tafel tests (Figure 5b).

**Figure 7:**
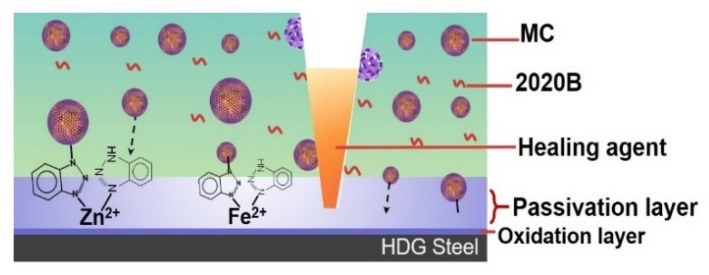
The effects of the dual functional GO microcapsules.

The effects of dual functional microcapsules are schematically shown in Figure 7. The self-healing properties were realized by the release of 2020A from the microcapsules and the curing reaction between 2020A and the dispersed 2020B. In addition, BTA on the shells of the GO microcapsules can either capture the metal ions in the coatings, or migrate to the metal/coating interface and form a passivation layer. The electrochemical process of corrosion reaction would be inhibited by the capture of metal ions and the formation of a passivation layer [29], which are independent of the breakage of the microcapsules.

## 4. Conclusions

Dual functional GO microcapsules featuring facile fabrication, high loading capacity, and robust encapsulation were prepared through self-assembly in Pickering emulsions with the assistance of BTA. The corrosion inhibitor BTA was carried on the microcapsule shells and the healing agent was encapsulated as the core materials, forming the albumen-yolk-like structure of the microcapsules. The loading capacity decreased from 90.5% to 80.9% with an increase in the BTA content in microcapsules, while the average diameter increased from 12.5 μm to 17.2 μm, indicating the increased thickness of the microcapsule shells. The physical and chemical interactions between BTA and GO helped the robust encapsulation of the healing agent. The GO microcapsules were dispersed in the WEP coating matrix. The *I_corr_* of the composite coatings containing 10 wt% MC-4 decreased for more than two orders of magnitudes compared with the neat WEP coatings. After scratching and healing for 48 h, the HE of the composite coatings reached over 99% when the content of MC-3 was higher than 10 wt%. We hope that our findings can extend the application of GO microcapsules to fulfil multi-functions and inspire various material selections by the self-assembly process.

## Figures and Tables

**Table 1:** The content of the Pickering emulsions for the preparation of the GO microcapsules.

Specimen	MC-0	MC-1	MC-2	MC-3	MC-4	MC-5
BTA (mg)	0	2	9	35	70	140
2020A (g)	1.4	1.4	1.4	1.4	1.4	1.4
GO (mL, 3.75 mg/mL)	5	5	5	5	5	5
Average diameter of the microcapsules (μm)	/	/	12.5	14.7	17.2	/
Loading capacity of the microcapsules (%)	/	/	90.5	87.7	80.9	/

**Table 2:** The atomic content of the elements detected by XPS.

Atomic Content (%)	C	O	N
GO	68.9	31.1	0
MC-2	80.2	19.1	0.7
MC-3	78.6	19.3	2.1
MC-4	74.7	21.7	3.6

**Table 3:** The electrochemical parameters for different coatings.

Coatings	Microcapsules/Content (wt%)	E_corr_ (mV)	*I_corr_* (A cm^−2^)	HE (%)
Intact WEP	0	−684	2.20 × 10^−10^	/
Intact	MC-2/10	−104	8.62 × 10^−11^	/
Intact	MC-3/10	158	4.59 × 10^−12^	/
Intact	MC-4/10	377	1.37 × 10^−12^	/
Scratched WEP	0	−930	1.68 × 10^−6^	0
Healed	MC-3/5	−430	8.75 × 10^−8^	94.8
Healed	MC-3/10	−334	4.71 × 10^−9^	99.7
Healed	MC-3/15	−279	2.27 × 10^−9^	99.9
